# Synergistic effects of carvacrol, α-terpinene, γ-terpinene, ρ-cymene and linalool against *Gardnerella* species

**DOI:** 10.1038/s41598-022-08217-w

**Published:** 2022-03-15

**Authors:** Lúcia G. V. Sousa, Joana Castro, Carlos Cavaleiro, Lígia Salgueiro, Mariana Tomás, Rita Palmeira-Oliveira, José Martinez-Oliveira, Nuno Cerca

**Affiliations:** 1 Centre of Biological Engineering (CEB), Laboratory of Research in Biofilms Rosário Oliveira (LIBRO), University of Minho, Braga, Portugal; 2 LABBELS –Associate Laboratory, Braga, Portugal; 3 Faculty of Pharmacy of the University of Coimbra, University of Coimbra, Coimbra, Portugal; 4 CIEPQPF, Department of Chemical Engineering, Faculty of Sciences and Technology, University of Coimbra, Coimbra, Portugal; 5 CICS-UBI, Health Sciences Research Center, Faculty of Health Sciences, University of Beira Interior, Covilhã, Portugal; 6 Faculty of Health Sciences, University of Beira Interior, Covilhã, Portugal; 7 CNC - Center for Neuroscience and Cell Biology, Center for Innovative Biomedicine and Biotechnology (CIBB), University of Coimbra, Coimbra, Portugal

**Keywords:** Microbiology, Diseases

## Abstract

Bacterial vaginosis (BV) is the most common vaginal infection affecting women worldwide. This infection is characterized by the loss of the dominant *Lactobacillus* community in the vaginal microbiota and an increase of anaerobic bacteria, that leads to the formation of a polymicrobial biofilm, mostly composed of *Gardnerella* spp. Treatment of BV is normally performed using broad-spectrum antibiotics, such as metronidazole and clindamycin. However, the high levels of recurrence of infection after treatment cessation have led to a demand for new therapeutic alternatives. *Thymbra capitata* essential oils (EOs) are known to have a wide spectrum of biological properties, including antibacterial activity. Thus, herein, we characterized two EOs of *T. capitata* and tested their antimicrobial activity as well as some of their main components, aiming to assess possible synergistic effects. Our findings showed that carvacrol and ρ-cymene established a strong synergistic antimicrobial effect against planktonic cultures of *Gardnerella* spp. On biofilm, carvacrol and linalool at sub-MIC concentrations proved more efficient in eliminating biofilm cells, while showing no cytotoxicity observed in a reconstituted human vaginal epithelium. The antibiofilm potential of the EOs and compounds was highlighted by the fact cells were not able to recover culturability after exposure to fresh medium.

## Introduction

Bacterial vaginosis (BV) is the most frequent gynaecological infection affecting women of reproductive age worldwide^1^. This infection is associated with vaginal discharge, unpleasant smell, an increase in vaginal pH, and the presence of clue cells^2,3^. Besides these common symptoms, BV has also been related to more serious health complications including preterm delivery^4^, pregnancy losses^5^, pelvic inflammatory disease^6^, and acquisition of sexually transmitted diseases^7^. BV is described by a decrease in the number of dominant lactic acid-producing species, normally *Lactobacillus* species, associated with healthy vaginal microbiota, and an overgrowth of strict and facultative anaerobic bacteria, such as *Gardnerella* spp., *Fannyhessea vaginae* (former *Atopobium vaginae*^8^), *Prevotella* spp., *Mobiluncus* spp., and other bacterial vaginosis-associated bacteria (BVAB)^9,10^. It is well known that BV involves the formation of a polymicrobial biofilm on the vaginal epithelial cells^11^, where bacterial species interact and develop the infection^12^. *Gardnerella* species have been observed as the most common bacteria present in cases of BV however, the role of this pathogen in the infection is still ambiguous^13^ since *Gardnerella* colonization does not always cause BV^14^. Recently a study proposed that what has been previously referred to as *Gardnerella vaginalis*, in fact, includes 13 different species of the genus *Gardnerella,* of which 4 have been described: *G. vaginalis*, *G. piotii*, *G. leopoldii,* and *G. swidsinskii*^15^. Studies regarding *Gardnerella* virulence features have suggested that some strains of *Gardnerella* have a preponderant role in initiating the colonization of the vaginal epithelium, outcompeting other vaginal pathogens^16^, followed by the formation of biofilm, affecting the progress of BV^12,17^. However, it is not currently clear if a specific *Gardnerella* species has higher virulence potential than others.

The treatment of BV is usually performed using the current recommended broad-spectrum antibiotics, namely metronidazole, and clindamycin^18^. However, a major issue related to BV is the fact that the treatment with conventional antibiotics often results in high recurrence rates^19^. This has been suggested to be a consequence of the inability of conventional therapies in eradicating the polymicrobial biofilm, which can be attributed to the presence of multidrug resistance species within the consortia^20,21^, which led to rapid regrowth after treatment cessation^22^. This poor treatment efficacy has led to an urgent need for new therapies to treat this infection^23^. As such, alternative therapies using natural products have been proposed, to overcome the resistance to antibiotics and prevent serious complications^24–26^.

Essential oils (EOs) are complex mixtures of natural volatile compounds extracted from aromatic plants, with low molecular weight, and have been used since ancient times for therapeutic purposes^27^. They have been recognized for their good antibacterial^28^, antifungal^29^, antioxidant^30^, and antiviral^31^ properties. In fact, regarding vaginal infections, several EOs have been tested with promising results^32–34^. However, one of the major disadvantages of using EOs is the fact that, as natural compounds, their compositions might differ between collections, possibly resulting in variations in their activities^35^. As such, studying the activity of the single compounds from EOs can provide important insights regarding their antimicrobial activity. The Portuguese flora is constituted by approximately 3800 species, of these about 600 *taxa* are aromatic plants^36,37^. Lamiaceae family is one of the most representative taxon^38^, with 29 genera represented in Portugal^39^. *Lavandula*, *Thymus,* and *Thymbra* are among the most important *genera* due to their wide use in Portuguese traditional medicines^37^. The genus *Thymbra* is represented in Portugal by only one species, *Thymbra capitata*^39^. *Thymbra capitata* (L.) Cav. is a plant widespread on the Mediterranean coast^40,41^ and its essential oil (EO) is characterized by high carvacrol amounts^40,42^. Previous studies demonstrated the biological activities of *T. capitata* EOs, such as antibacterial^43^, antifungal^41^, and antioxidant^44^ effects against different microorganisms. We recently demonstrated the potential of *T. capitata* EO against *Gardnerella* spp. growing planktonically and as biofilm^45^.

In this work, we characterized two samples of *T. capitata* EO, collected in different locations and tested their antimicrobial activity against the 4 described *Gardnerella* species. We also assessed potential synergism between some of the EO main components, aiming to develop a future topical application against *Gardnerella* biofilms.

## Results

### Essential oils composition

The results from the analysis of EOs revealed that the composition of the two oils was similar, with both EOs being mainly composed of carvacrol (73.9–80.0%) and its biogenetic precursors, γ-terpinene (3.4–7.4%) and *p*-cymene (4.1–4.9%). The full composition of both oils is detailed in Table 1.

**Table 1:** Constituents of two samples of essential oils from *Thymbra capitata*.

Compound*	RI SPB-1^a^	RI SW 10^b^	Sample Ben. (%)	Sample Car. (%)
α-Thujene	922	1029	0.4	1.4
α-Pinene	930	1030	0.3	0.6
Oct-1-en-3-ol	956	1440	t	0.1
Sabinene	964	1128	t	t
β-Pinene	970	1118	0.3	0.1
Myrcene	980	1161	1.1	1.8
α-Phellandrene	997	1171	0.1	0.2
3- Carene	1003	1155	t	0.1
α-Terpinene	1010	1187	0.4	1.5
*p*-Cymene	1011	1275	4.1	4.9
Limonene	1020	1206	0.1	0.2
β-Phellandrene	1020	1215	0.1	0.2
Z-β-Ocimene	1025	1235	t	t
E-β-Ocimene	1035	1253	t	0.1
γ-Terpinene	1046	1249	3.4	7.4
*trans*-Sabinene hydrate	1050	1459	0.4	0.6
Cymenene	1073	1440	0.1	0.1
*cis*-Sabinene hydrate	1080	1544	0.2	0.1
Linalool	1081	1543	1.0	0.3
*trans*-*p*-2-Menthen-1-ol	1122	1623	t	t
Borneol	1144	1695	0.1	0.2
Terpinene-4-ol	1158	1597	0.7	0.7
*trans*-Dihydrocarvone	1167	1602	t	t
α-Terpineol	1169	1692	0.1	0.1
Neral	1214	1679	t	t
Geraniol	1233	1842	t	0.1
Geranial	1242	1730	0.1	0.1
Thymol	1268	2183	0.1	0.2
Carvacrol	1275	2212	80.0	73.9
*E*-Caryophyllene	1408	1590	1.9	3.4
Aromadendrene	1425	1600	t	t
α-Humulene	1443	1662	0.1	t
*Allo*-aromadendrene	1445	1636	0.1	0.1
Bicyclogermacrene	1481	1726	0.1	0.1
Caryophyllene oxide	1557	1968	0.2	0.2
Total identified			95.8	99.0

*Compounds listed in order of elution in the polydimethylsiloxane column (SPB-1 column).

^a^RI SPB 1: GC retention indices relative to C9-C23 n-alkanes on the SPB-1 column.

^b^RI SW 10: GC retention indices relative to C9-C23 n-alkanes on the Supelcowax-10 column. t = traces ≤ 0.05%.

### Minimal inhibitory concentration and minimal lethal concentration

**Figure 1:**
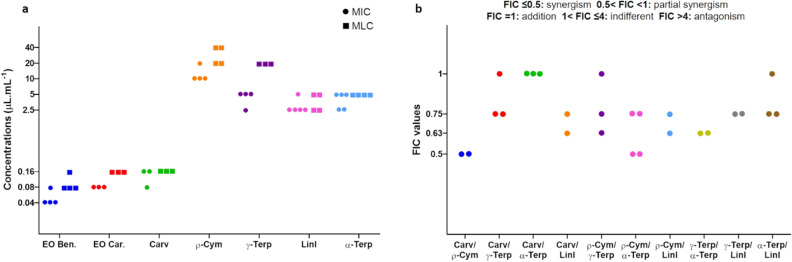
Antimicrobial activity of two EOs from *Thymbra capitata*, carvacrol, ρ-cymene, γ-terpinene, linalool, and α-terpinene against *Gardnerella* sp. UM241. (**a**) Determination of the minimum inhibitory and minimum lethal concentration. (**b**) Results from checkerboard assays of combinations between the selected individual compounds from the EO. Individual points represent distinct checkerboard combinations. In both panels, each point represents the results from independent assays.

It has been shown that the biological effects of EOs are possibly due to the combined effects of their components, particularly the main ones^46^. In order to recognize the antimicrobial activity of some of these compounds, that are commercially available, the values of minimal inhibitory concentration (MIC) and minimal lethal concentration (MLC) were first determined. As shown in Fig. 1a, both the EOs had similar antimicrobial activity against the selected *Gardnerella* isolate. Of the tested individual components, carvacrol was by far the most potent, with a MIC value very similar to the whole EO. Despite having similar high concentrations in both EO, ρ-cymene and γ-terpinene had a fourfold difference in the MIC values, while linalool, which was present in a lower concentration, reported a similar antimicrobial activity as γ-terpinene. Interestingly, for all the active compounds, the values of MLC were almost coincident with the values of MIC.

### Combined effects of individual compounds of EOs

It has been previously shown that the combined effect of some individual compounds can enhance their antimicrobial activities^47,48^. Taking into consideration the MIC determinations, we assessed possible synergism between the individual components tested. As shown in Fig. 1b, different effects were observed wherein combinations of carvacrol and ρ-cymene resulted in the highest synergistic activity. No antagonistic effects were observed.

### Activity of EOs and compounds on a *Gardnerella* biofilm

**Figure 2:**
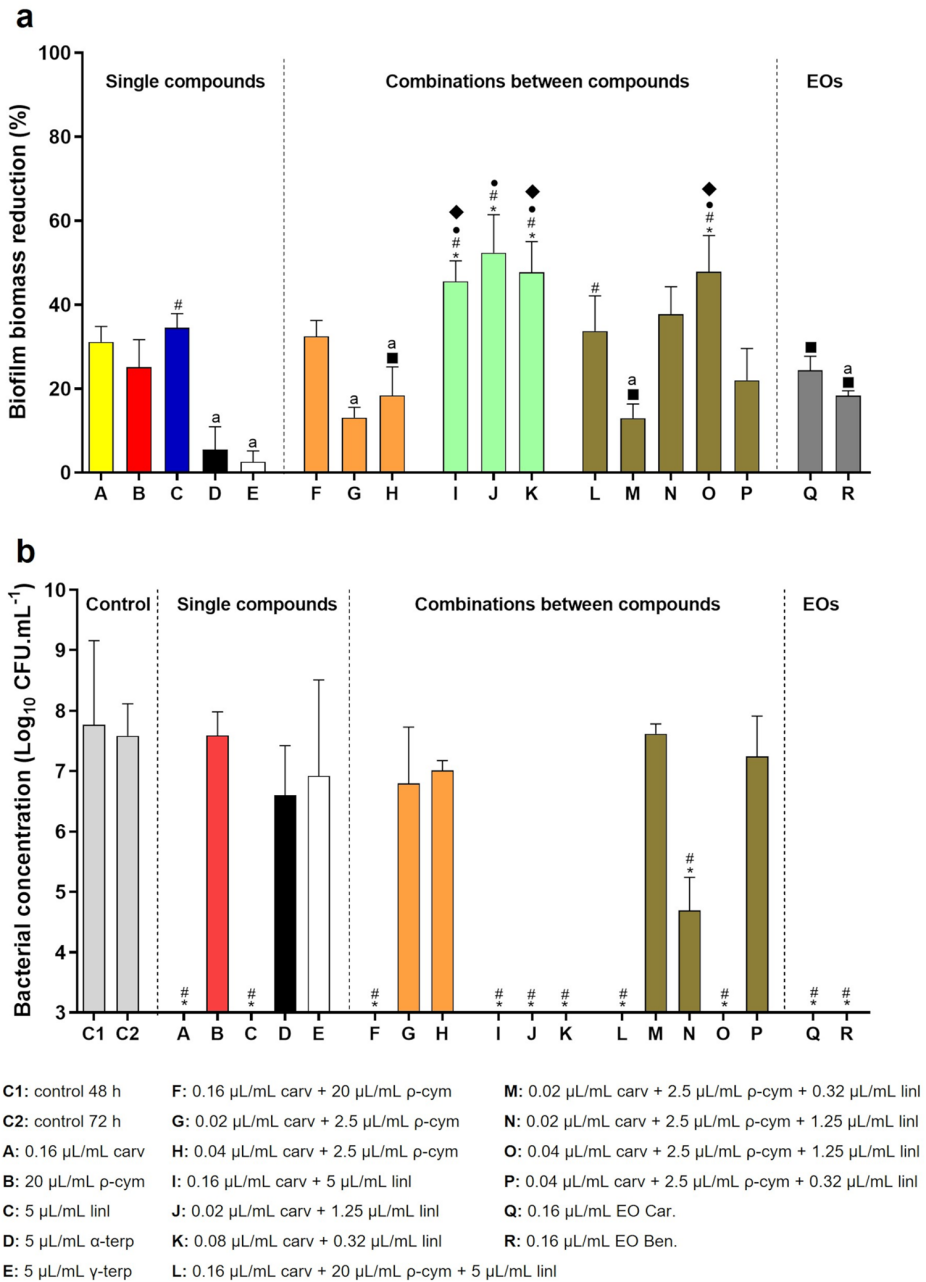
Activity of single components, combinations between compounds and EOs from *Thymbra capitata* on a 48 h *Gardnerella* sp. UM241 biofilm. (**a**) Effect on biofilm biomass reduction. Results are presented in mean percentage (%) of biofilm biomass reduction. (**b**) Effect on biofilm cells culturability. Results are represented as mean of Log (CFU.mL^−1^) with a limit of detection of Log=3.﻿ Primary colors were selected to represent the individual components and the mixtures represent the color combination of the individual components. Statistical analysis was performed using one-way ANOVA (n ≥ 3). In panel (**a**), differences are showed, when p < 0.05, by asterisk symbol when comparing with 5 µL/mL α-terp; hash symbol when comparing with 5 µL/mL γ-terp; bullet symbol when comparing with 0.02 µL/mL carv + 2.5 µL/mL ρ-cym; filled square symbol when comparing with 0.02 µL/mL carv + 1.25 µL/mL linl and filled diamond symbol when comparing with 0.02 µL/mL carv + 2.5 µL/mL ρ-cym + 0.32 µL/mL linl. All conditions are statistically different from the 48 h-biofilm control except for the cases marked (**a**). In panel (**b**), differences are represented by asterisk symbol when comparing with control 48 h and hash symbol when comparing with control 72 h, when p < 0.05.

Since BV is well-known to be associated with biofilm formation, mainly constituted by *Gardnerella*^11^, we further tested the EO and some of their components' anti-biofilm activity. As shown in Fig. 2a, α- and γ-terpinene had no effect in reducing the biomass of the biofilm, despite their antimicrobial activity assessed against planktonic cultures. Carvacrol, ρ-cymene, and linalool add a similar effect at either of the two EOs tested. Interestingly, several combinations of carvacrol and linalool, at sub-mic concentrations, revealed a significantly higher ability to reduce the biofilm biomass, as compared with the MLC concentrations of the EOs. The addition of ρ-cymene did not enhance the carvacrol and linalool synergistic effect. Interestingly, when assessing cell culturability, the total reduction of CFU’s was observed for both EOs, carvacrol, linalool, or any mixture of these 2 components (Fig. 2b).

The effect of DMSO on *Gardnerella* sp. UM241 cells culturability was also evaluated and the results are described in Supplementary Fig. 1. DMSO did not show any negative effect on cells viability at the different concentrations tested.

**Figure 3:**
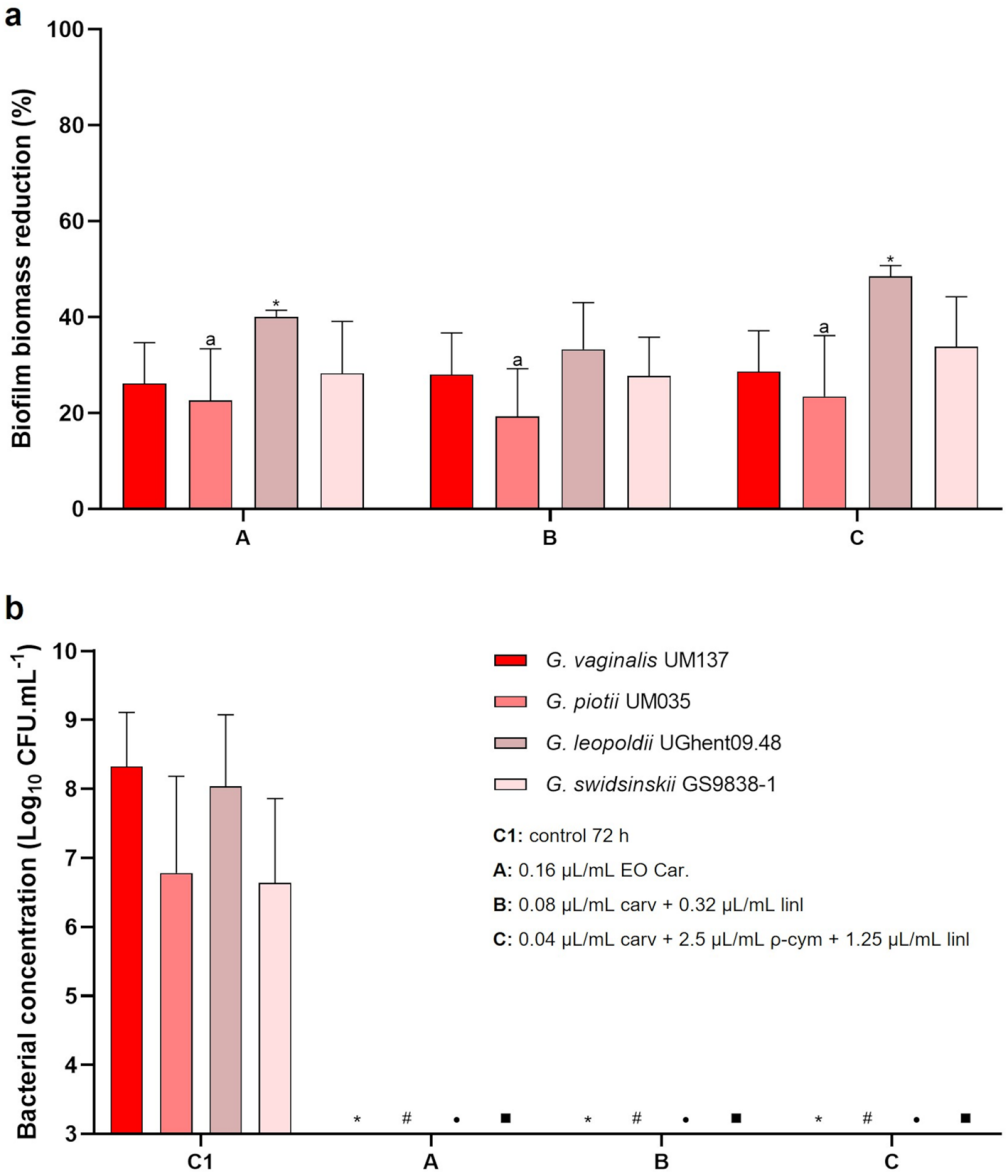
Activity of EO Car. and two combinations among compounds on a 48 h biofilm of *G. vaginalis* UM137, *G. piotii* UM035, *G. leopoldii* UGent 09.48 and *G. swidsinskii* GS 9838–1. **a**: Effect on biofilm biomass reduction. Results are presented in percentage (%) of biofilm biomass reduction. Error bars represent s.e.m. Statistical differences were found for all the conditions when compared with the 72 h-biofilm control, except for *G. leopoldii* where no differences were found (**a**) (t-test, n = 4). Statistical differences were found in *G. leopoldii,* between conditions A and C (asterisk symbol) (t-test, n = 4). (**b**) Effect on biofilm cells culturability. Results are represented as mean of Log (CFU.mL^−1^) with a limit of detection of Log = 3. Error bars represent s.d. Statistical analysis was performed using t-test (n = 4). Differences are represented when p < 0.05, when compared with control 72 h of G. *vaginalis* UM137 (asterisk symbol), *G. piotii* UM035 (hash symbol), *G. leopoldii* UGent 09.48 (bullet symbol) and *G. swidsinskii* GS 9838-1 (filled square symbol).

Other four *Gardnerella* species were used to assess the activity of all components on biofilms. We have chosen the conditions that were able to eliminate the growth of *Gardnerella* sp. UM241, namely the EO sample from Carvoeiro, the combination between carvacrol and linalool with the lowest amount of linalool, and a combination of 3 selected compounds (at their lowest concentrations tested). The results are shown in Fig. 3. The capacity of the compounds to reduce biofilm biomass (Fig. 3a) was similar to that observed when tested in *Gardnerella* sp. UM241 biofilm. When evaluating the results of bacterial culturability (Fig. 3b) it is possible to verify the inhibition of growth for all the species in all the tested conditions.

### Effect of EOs and compounds on *Gardnerella* biofilm structure and viability

**Figure 4:**
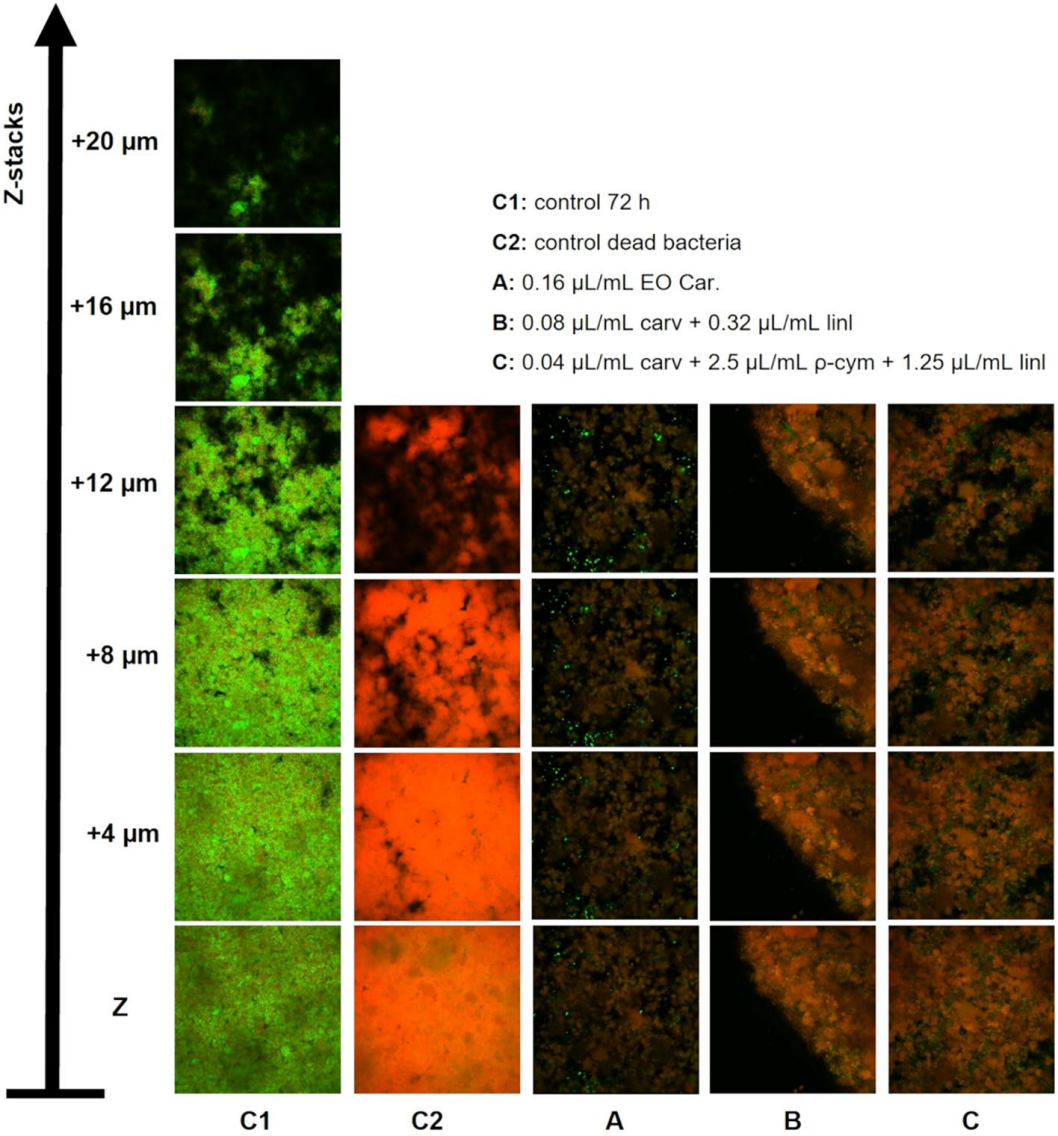
Representative images of CLSM analysis of *G. vaginalis* UM137 72 h biofilm after the activity of EO Car. and some specific combinations using LIVE/DEAD staining. The images were acquired using an objective of 10 × and z-stacks of 4 µm.

**Figure 5:**
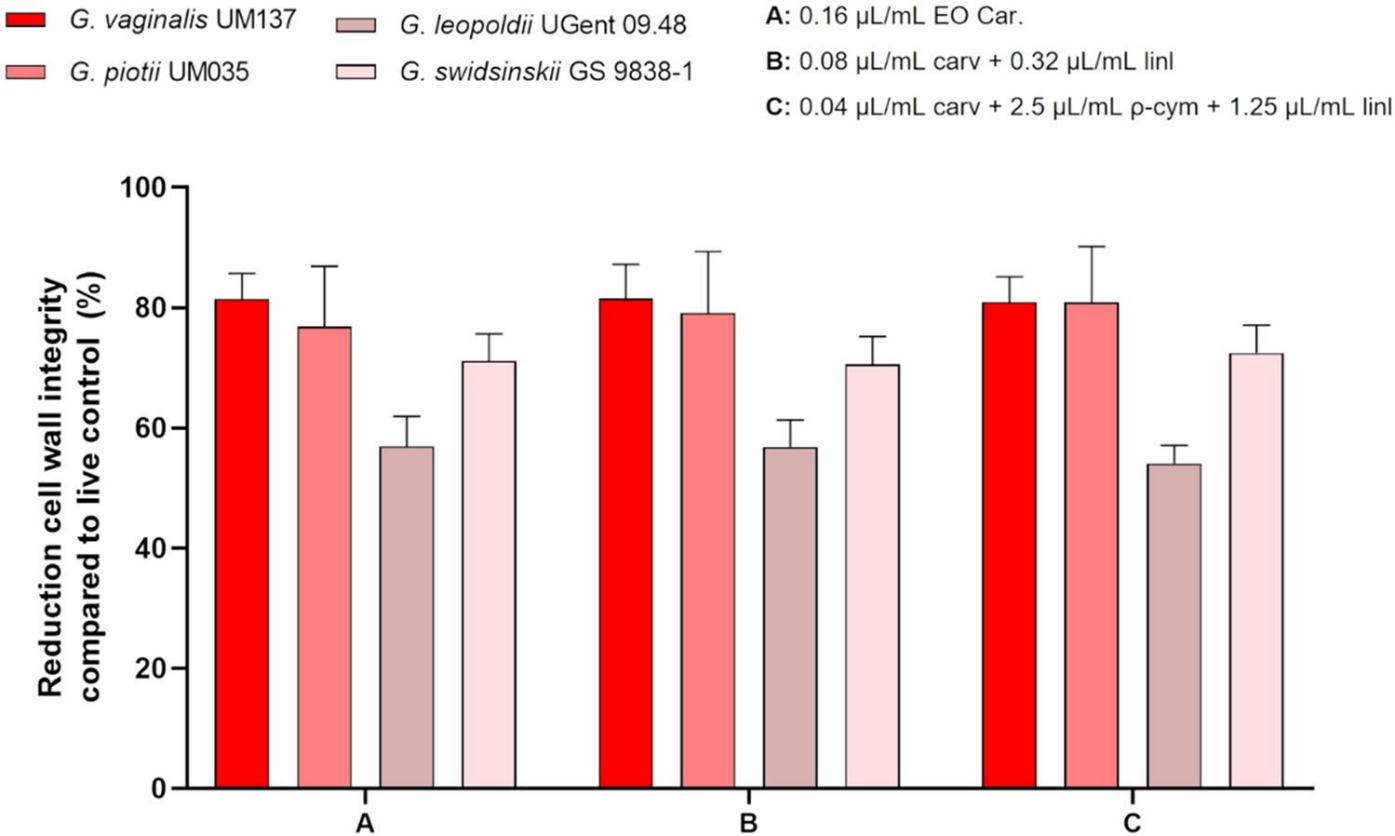
Results from LIVE/DEAD quantitative analysis of *G. vaginalis* UM137, *G. piotii* UM035, *G. leopoldii* UGent 09.48 and *G. swidsinskii* GS 9838-1 48 h biofilms after the activity of EO Car. and two combinations of compounds. The results are shown in percentage of reduction of cell wall integrity compared to live control. Error bars represent s.d. No statistical differences were found when using a t-test (n = 3) comparing the different conditions within the same tested species. All conditions are statistically different from the control.

Since we observed a total reduction of cultivable bacteria without completely removing the biofilm structure, we decided to analyze the biofilm structure after the antimicrobial challenge, by using LIVE/DEAD staining and CSLM observation. As shown in Fig. 4, the big majority of biofilm cells treated with the EO or the two most promising combinations of components, presented a damaged cell wall, similar to the dead cells from biofilm control. Nonetheless, some cells still keep an intact cell wall, but they cannot be recovered (Fig. 3b), which suggests the presence of viable but not cultivable cells (VBNC)^49^. To confirm the CLSM observation and to quantitatively determine the % of viable or dead cells in biofilms formed by the 4 species of *Gardnerella*, after antimicrobial challenge, we performed a fluorimeter assay, based on LIVE/DEAD staining. As shown in Fig. 5, despite no culturable cells being recovered, 20 to 40% of biofilm cells maintained their cell wall intact, being *G. leopoldii* UGent 09.48 the strain with higher tolerance to the tested antimicrobial agents.

### Recovery of *Gardnerella* spp. cells culturability

**Figure 6:**
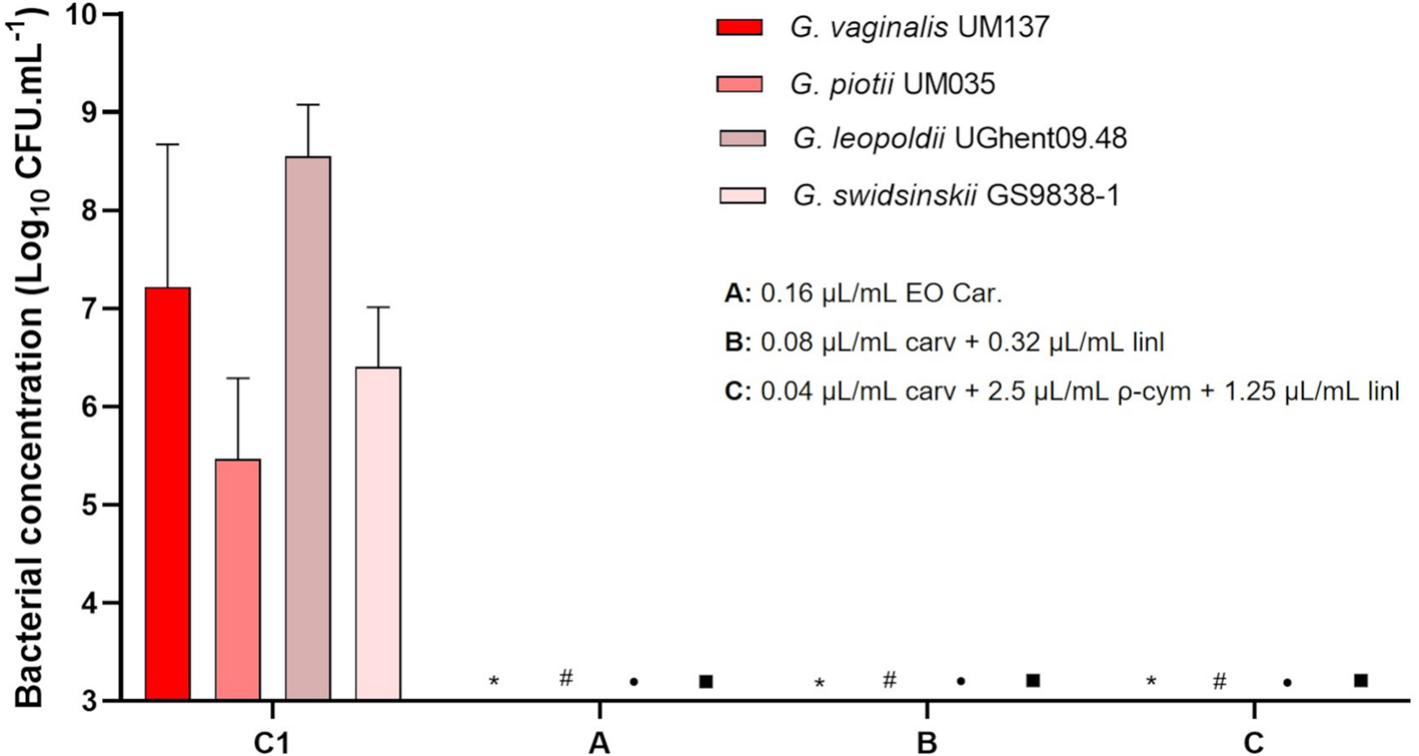
Analysis of biofilm cells culturability recovery of different *Gardnerella* species after the effect of EO Car. and specific combinations of compounds. The experiments were performed after 96 h of biofilm formation with sBHI medium. Results are represented as mean of Log (CFU.mL^−1^) with a limit of detection of Log = 3, and the error bars represent s.d. Statistical analysis was performed using a t-test (n = 4). Differences are represented when p < 0.05, when compared with control of *G. vaginalis* UM137 (asterisk symbol), *G. piotii* UM035 (hash symbol), *G. leopoldii* UGent 09.48 (bullet symbol) and *G. swidsinskii* GS 9838-1 (filled square symbol). C1: control (48 h-biofilm + 24 h sBHI + 24 h sBHI); A: 0.16 µL/mL EO Car; B: 0.08 µL/mL carv + 0.32 µL/mL linl; C: 0.04 µL/mL carv + 2.5 µL/mL ρ-cym + 1.25 µL/mL linl.

With the detection of a variable percentage of VBNC in all tested species, we performed another experiment, wherein we attempt to recover the culturability of the biofilm cells, by allowing the remaining biofilm to be incubated in fresh sBHI, after removing the antimicrobial agents. As shown in Fig. 6, no recovery of culturability was detected, after 24 h of incubation in fresh medium.

### Colonization of reconstructed human vaginal epithelium

**Figure 7:**
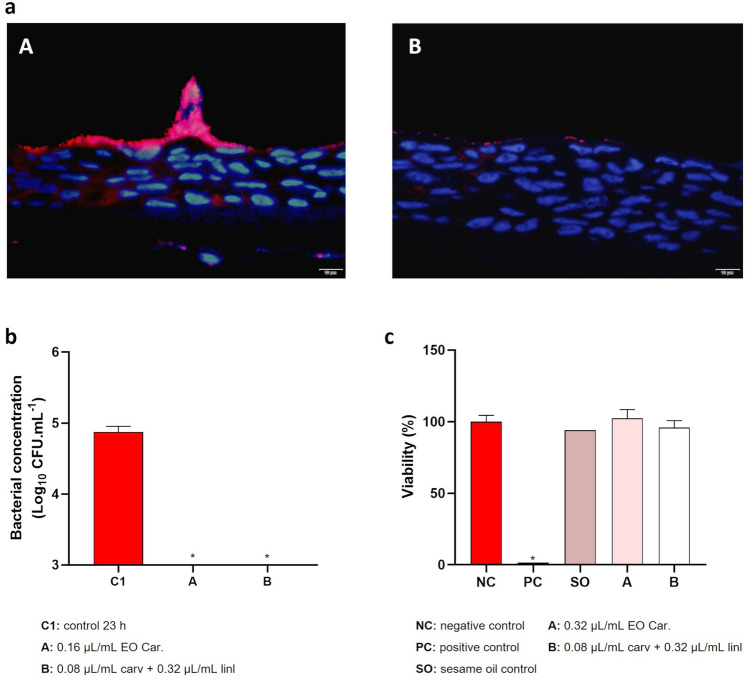
Colonization of reconstituted human vaginal epithelium and cytotoxicity of *T. capitata* EO. (**a**) Microscopic images of reconstituted human vaginal epithelium after 23 h of colonization with *G. vaginalis* UM137 (A) and after 9 h of *G. vaginalis* UM137 colonization with 14 h of contact with EO Car. (B). (**b**) Results of cells culturability after colonization of vaginal epithelium. Results are represented as mean of Log (CFU.mL^−1^) with a limit of detection of Log = 3. Error bars represent s.d. Statistical analysis was performed using t-test (n = 3 for control and EO assay; n = 2 for carvacrol + linalool assay). Differences are represented by * when comparing with control 23 h, when p < 0.05. (**c**) Results of cytotoxicity of EO and combination carvacrol + linalool on the vaginal tissue are the mean of percentage (%) of viability. Error bars represent s.d. Statistical analysis was performed using a t-test (n = 4 for EO assay; n = 3 for controls and carvacrol + linalool assay). Differences are represented by * when comparing with the negative control. Statistical analysis did not include sesame oil control (n = 1).

To assess the antimicrobial potential of the EO or carvacrol + linalool, *G. vaginalis* early-stage biofilms were grown on a human vaginal epithelium tissue, using a chemically defined medium that simulates genital tract secretions. The biofilms were then challenged at the EO MLC concentration or the compounds at the concentrations where synergism was observed. As represented in Fig. 7a, *G. vaginalis* not exposed to the antimicrobial agents was able to form a biofilm on the vaginal epithelium, and the biofilm was drastically reduced after the antimicrobial challenge. Like the previous experiments, no culturable cells were recovered after the antimicrobial challenge (Fig. 7b). Interestingly, at this concentration, neither the EO nor the carvacrol + linalool combinations demonstrated significant cytotoxicity (Fig. 7c).

## Discussion

The increasing levels of bacterial resistance associated with commonly used antimicrobial agents are one of the problems that most affect public health^50^. In BV, increased resistance to metronidazole and clindamycin have been reported^51–53^, and this led to seek for new alternatives of treatment^54^. As far as we are aware, the use of EOs as alternatives for the treatment of vaginal infections is still limited, however, some EOs have been tested on *Candida* species associated with vaginal candidiasis^55,56^, in BV^45,57,58^, trichomoniasis^59^ or other vaginal infection-related pathogens^60^. One possible criticism of the utilization of EO as a reliable therapeutically option is the variability that is often observed between different batches of the same EO, which can affect its properties and biological activities^61^. Thus, it is extremely important to characterize the essential oils to ensure their chemical homogeneity. According to our results, the EOs analyzed were very homogeneous, characterized by high contents in carvacrol. Interestingly, the antimicrobial potential of the two EOs was also very similar. Furthermore, assessing the antimicrobial activity of single compounds or mixtures of compounds present in the EOs, has the potential to develop a more standardized product, despite having increased costs of production. In this study, we decided to study single compounds and combinations of compounds from *T. capitata* EO, in order to find a composition that has good antimicrobial activity against biofilms formed by *Gardnerella* spp.

Due to the wide-ranging biological properties attributed to *T. capitata* EO, several studies have been conducted to assess the antibacterial activity of *T. capitata* EO and carvacrol, and to less extent the activity of their compounds^62^. As a result, it has been shown that the antimicrobial activity of EOs is dependent on their chemical compositions^63,64^. The high content of carvacrol, a phenolic monoterpene, in *T. capitata* EO is pointed to be largely responsible for the antimicrobial activity^65^. Not surprisingly, herein, carvacrol also presented the highest antimicrobial activity of the five compounds tested. It has also been described that monoterpenes have low antimicrobial activity and are usually ineffective as antimicrobial agents when used alone^66,67^, as also observed in this study. Nevertheless, components present in small amounts in EOs can still play an important role in antimicrobial activity due to the possible synergistic effects^68,69^.

It has been previously shown that interactions between major and minor constituents can have a critical influence on global antimicrobial activity^27^. The most common interactions between different components include synergistic, antagonistic, additive, and indifferent effects^70^. Noticeably, the strongest effect happened in the combination of carvacrol and ρ-cymene resulting in synergism. This effect is expected to result from the interaction of ρ-cymene with the lipidic membrane of the cells causing the expansion of the membrane, which facilitates and increases the transportation of carvacrol into the cells^66,71^. Other studies already reported this synergistic effect between carvacrol and ρ-cymene, particularly against *Listeria monocytogenes*^72^ and *Escherichia coli*^73^.

Interestingly, when testing the antimicrobial activity against biofilms, α- and γ-terpinene had no detectable effect, and while ρ-cymene was able to reduce ~ 30% of the total biomass, its effect on bacterial culturability was neglectable. Furthermore, despite carvacrol + ρ-cymene having a better synergistic effect against planktonic cultures, on biofilms, the most active combination was carvacrol + linalool. This last combination was more effective in reducing the total biomass of the biofilm (~ 50%) than the whole EO (~ 30%). This further demonstrates that biofilm cultures susceptibility to antimicrobials cannot be predicted from the standardized MIC determinations^74,75^. Despite not being able to completely eradicate the biofilm biomass, cell culturability was significantly affected, with no colony detected in many of the tested conditions. A similar effect was also reported with *T. capitata* EO against a biofilm of *Candida glabrata* where despite the low reduction of biofilm biomass, the cells were not metabolic active, remaining inactive or dead^41^. In another study with *Staphylococcus aureus* and *E. coli* biofilms, five different EOs also promoted the reduction of cells metabolic activity inpre-established biofilms and even the complete elimination of viability in some of the cases^76^.

To further understand the impact of the EO and some of the most promising mixtures, we also performed a resuscitation attempt^77^, whereby stimulating the cells without the antimicrobial stress, they can recover the full active metabolic state and culturability. However, in our tested conditions, no cell recovery was observed. This observation suggests that these antimicrobial agents have a lasting effect on *Gardnerella*, and this might improve the recurrence rates currently observed after treatment with metronidazole or clindamycin^78,79^.

Herein, we also determined the feasibility of using reconstituted vaginal epithelial tissue, to establish a *Gardnerella* spp. biofilm. While this vaginal model has been used before to study *Candida* infection^80^ or vaginal dryness^81^, as far as we are aware, this is the first study that reports *Gardnerella* spp*.* colonization in this model. By using a PNA-FISH specific probe, we were able to observe intact biofilms formed on the vaginal epithelium. Interestingly, after the antimicrobial challenge, most of the bacterial cells were killed, and the small clusters of cells observed were non-cultivable. Importantly, at the tested concentration used herein, we did not observe any significant cytotoxicity in the vaginal tissue.

In summary, this study revealed the potential of *T. capitata* EO and some of its components to be used as potential new therapeutic agents in the treatment of BV. Nonetheless, this study has some limitations and further work should be performed to test these components on multi-species biofilms, that better mimic what occurs in BV^82^. Furthermore, it would be important to assess their activity against *Lactobacillus* species common colonizers of the healthy vaginal microbiota. In addition, similar to what is described in other EOs^83^, it would be also important to analyze the effect of the tested components with conventional antibiotics. Apart from it, there are some limitations in the methods, namely in the biofilm biomass quantification by CV, since it is known that one of the disadvantages of this method is the lack of reproducibility^84^.

## Materials and methods

### Plant material and commercial compounds

Aerial parts of *T. capitata* plant were collected at the flowering stage in two different locations of the Algarve region (south of Portugal): Benagil (sample Ben., Benagil beach: 37°05′13.1″N 8°25′35.5″W) and Carvoeiro (sample Car., Carvoeiro: 37°05′33.9″N 8°27′55.5″W), to obtain oils from two different populations. The collection site access has been approved by Mr. Daniel Prado, the local owner. Dr. Jorge Paiva, a taxonomist at the University of Coimbra, confirmed species authenticity and plant names were checked with https://www.theplantlist.org. Voucher specimens were included in the Herbarium of the University of Coimbra (COI), with the accession number Salgueiro 19, and in the Herbarium of the Faculty of Pharmacy of UC, with the accession numbers Salgueiro 135 and Salgueiro 196. All plant collection procedures complied with relevant institutional, national and european legislation. Furthermore, five commercial compounds, including the main compound of the EOs (carvacrol (carv), γ-terpinene (γ-terp), ρ-cymene (ρ-cym), Sigma-Aldrich, Missouri, USA). Other minor monoterpenes (α-terpinene (α-terp) and linalool (linl), Sigma-Aldrich), that previously showed effective antimicrobial activity^85–87^, were also tested against *Gardnerella* species.

### Essential oil extraction and analysis

The EOs were isolated by hydrodistillation for 3 h, using a Clevenger-type apparatus according to the European Pharmacopoeia^88^. The oils were preserved in a sealed vial at 4ºC. The compositions of the *T. capitata* EO were established by the combination of gas chromatography with FID detectors (GC-FID) and gas chromatography-mass spectroscopy (GC/MS) analysis as previously reported^89^. GC-FID analysis was performed in a Hewlett-Packard 6890 gas chromatograph (Agilent Technologies, Palo Alto, CA, USA) set with a single injector and two flame ionization detectors (FID). A divider (Agilent Technologies, part no. 5021-7148) was used for simultaneous sampling on two fused silica capillary columns: a SPB-1 (polydimethylsiloxane 30 m × 0.20 mm i.d., film thickness 0.20 µm) and a SupelcoWax-10 (polyethyleneglycol 30 m × 0.20 mm i.d., film thickness 0.20 µm). Oven temperature program: 70–220 °C (3 °C min^−1^), 220 °C (15 min); injector temperature: 250 °C; mobile phase: helium, with flow adjusted to maintain a linear velocity of 30 cm s^−1^; split ratio 1:40; detectors temperature: 250 °C. GC–MS analysis was performed in an Hewlett-Packard 6890 gas chromatograph interfaced with an Hewlett-Packard mass selective detector MSD 5973 (Agilent Technologies). An HP1 (Agilent Technologies) fused silica column (polydimethylsiloxane 30 m × 0.25 mm i.d., film thickness 0.25 µm) was used. GC parameters as described above; interface temperature: 250 °C; MSD parameters: interface temperature: 250 °C; MS source temperature: 230 °C; MS quadrupole temperature: 150 °C; ionization energy: 70 eV; ionization current: 60 µA; scan range: 35–350 units; scans s^−1^: 4.51.

The oils components were identified by considering, concurrently: (i) the acquired retention indices on two columns with different phases SPB-1 (polydimethylsiloxane) and SupelcoWax-10 (polyethyleneglycol) determined by linear interpolation relative to the retention times of C8–C23 of n-alkanes and compared with reference data from authentic products (available in the laboratory database of the Faculty of Pharmacy, University of Coimbra) and literature data^90^; (ii) the acquired mass spectra compared with reference data from the laboratory database, the Wiley/NIST library^91^ and literature^92^. The relative amount of each component was estimated from GC peaks areas without any correction regarding FID responses.

### Bacterial growth conditions

Various strains from the genus *Gardnerella* were analyzed in this study. *Gardnerella* sp. UM241, previously recovered from patients diagnosed with BV^93^, *G. vaginalis* UM137, *G. piotii* UM035, *G. leopoldii* UGent 09.48, and *G. swidsinskii* GS 9838-1, previously identified by MALDI-TOF^15,94^, were used herein. The strains were kept frozen in Brain Heart Infusion medium (BHI, Liofilchem, Roseto degli Abruzz, Italy) with 23% (v/v) of glycerol (Panreac, Barcelona, Spain) and plated in Columbia Blood Agar (CBA) [Columbia Base Agar medium (Liofilchem) supplemented with 5% (v/v) of defibrinated horse blood (Oxoid Ltd, Hampshire, UK)] and incubated for 48 h at 37 °C and 10% CO_2_. For each experiment, planktonic cells were grown in BHI supplemented (sBHI) with 2% (w/v) gelatin (Liofilchem), 0.1% (w/v) starch (Panreac) and 0.5% (w/v) yeast extract (Liofilchem) and incubated for 24 h at 37 °C and 10% CO_2_ (CO_2_ Incubator MCO-18AC, Panasonic, Bracknell, UK).

### Determination of the minimal inhibitory concentration and minimal lethal concentration

The MIC and MLC of both EOs, as well as five individual components of the oil, were evaluated by the macrodilution method, using the isolate *Gardnerella* sp. UM241, as described previously^45^ with some modifications. Briefly, each component was dissolved in the same proportion (1:1) in dimethyl-sulfoxide (DMSO, Scharlau, Barcelona, Spain) to improve its solubility. Therefore, serial dilutions in glass test tubes were prepared in sBHI to obtain a range of concentrations between 0.04 and 40 µL/mL in the experiments with the compounds. Regarding the experiments with the EOs, a range of concentrations between 0.02 and 2.5 µL/mL was tested. Afterwards, 500 µL of the bacterial inoculum, adjusted to the concentration of 10^6^ colony forming units (CFU)/mL, was added to each tube performing a total volume of 1 mL. Positive control with DMSO at the highest concentration used in the dilutions was included. A second positive control was used with bacterial suspension without any compounds or EOs. The negative control only included sBHI medium. After 48 h of incubation at 37 °C and 10% CO_2_, the MIC value was determined by reading the optical density (OD) of all the dilutions at 620 nm. MLC was determined by plating 10 µL of each dilution on CBA plates and defined as the lowest concentration where no growth was detected. Each experiment was repeated at least three independent times.

### Determination of the fractional inhibitory concentration by the checkerboard method

The checkerboard method was performed to study the interactions and the resultant effect from the combination of pairs of compounds from *T. capitata* against the isolate *Gardnerella* sp. UM241. Serial dilutions of each compound were prepared as described above in a range of concentrations appropriated to include the value of MIC for each compound. The experiments were performed on glass tubes for a final volume of 1 mL. For one of the compounds, serial dilutions were added along the lines and for the other compound were added along with the columns. Thereafter, 500 µL of a bacterial inoculum, prepared as described above and adjusted to the concentration of 10^6^ CFU/mL, was added to all the tubes. Then, the tubes were incubated for 48 h at 37 °C and 10% CO_2_. To analyze the effect of the interactions between the compounds, the OD of the content of each tube was measured at 620 nm, and the value of the fractional inhibitory concentration (FIC) index was determined according to the lowest concentrations in which bacterial growth was not observed. The FIC was calculated as previously described^95^, by $\mathrm{FIC}=\mathrm{FIC A}+\mathrm{FIC B}$, where $\mathrm{FIC A}=\frac{\mathrm{MIC A }\left(\mathrm{combination}\right)}{\mathrm{MIC A }\left(\mathrm{alone}\right)}$ and $\mathrm{FIC B}=\frac{\mathrm{MIC B }(\mathrm{combination})}{\mathrm{MIC B }(\mathrm{alone})}$^96^. The effect was considered synergistic when FIC was ≤ 0.5, partial synergistic when FIC was > 0.5 and < 1, additive when FIC = 1, indifferent when FIC was > 1 and ≤ 4, and antagonistic when FIC was > 4^97^. In each assay, the lowest value of FIC was selected, and the FIC of each combination was presented by the range of values from all assays. For each combination, experiments were performed at least two independent times.

### Biofilm formation and activity of EOs and components from *T. capitata*

Biofilm formation experiments were performed as previously described^98^ for all the *Gardnerella* strains used in this study. Briefly, the cell concentration of 24 h-old cultures was assessed by OD at 620 nm and the inoculums were further diluted to obtain a final concentration of 10^8^ CFU/mL. Biofilms were incubated in 96-well flat-bottom tissue culture plate (Orange Scientific, Braine-l’Alleud, Belgium) in 100 µL of sBHI for 48 h at 37 °C and 10% CO_2_, with fresh medium replacement after 24 h. The antimicrobial challenge occurred for another 24 h at the required concentrations of compounds. In a first assessment, the biofilm of *Gardnerella* sp. UM241 was challenged with the EOs at MLC concentration and single components at the highest MIC concentration. The sub-MIC concentrations for combinations between compounds were chosen based on the results from checkerboard assays, where some type of interaction between the components was observed. The following experiments were performed for the other 4 species of *Gardnerella* spp. and the conditions tested were EO Car. at 0.16 µL/mL, and the combinations of 0.08 µL/mL carvacrol + 0.32 µL/mL linalool and 0.04 µL/mL carvacrol + 2.5 µL/mL ρ-cymene + 1.25 µL/mL linalool. Two controls were included in each experiment. A positive control included a biofilm that was incubated for a total of 72 h without any antimicrobial challenge. Another control included the final incubation with DMSO in the maximum concentration used to dissolve the compounds. Biofilms were then quantified after 72 h of incubation. Each experiment was repeated at least three independent times with seven technical replicates.

### Biofilm biomass quantification by crystal violet staining method

Biofilm quantification by crystal violet (CV) was performed as previously reported^99^. Briefly, after the washing and fixation step, 100 µL of 1% (w/v) CV solution (Acros Organics, New Jersey, USA) was added to each well for 20 min. The CV solution was removed, and the biofilms were washed twice with 200 µL of H_2_O. Finally, 150 µL of 33% (v/v) acetic acid (Thermo Fisher Scientific, Lenexa, KS, USA) was dispensed into each well and the OD was measured at 595 nm using a microplate reader. The reduction in biofilm biomass was determined by comparing the values of OD of each condition with the OD values of the positive control without DMSO.

### Biofilm cells culturability analysis by colony-forming units

Biofilm formation was performed as described above, with the difference that 24-well flat-bottom tissue culture plates (Orange Scientific) were used, in which a coverslip (Thermo Scientific™ Nunc™ Thermanox™ Coverslips) was distributed in each one of the wells. After that, 1 mL of 24 h-bacterial inoculum, adjusted to the concentration of 10^8^ CFU/mL, was dispensed in each well and the plate was incubated at 37 °C and 10% CO_2_ with replacement by the fresh medium at 24 h. After 48 h, the growth medium was removed and the coverslips were transferred to a new 24-well plate. Thereafter, 300 µL of the desirable compounds in sBHI were dispensed into the wells and the plate was incubated for more 24 h at 37 °C and 10% CO_2_. After that time, the medium was removed, and each coverslip was washed with NaCl 0.9% (w/v). 300 µL of fresh sBHI was added to the wells and the biofilm was mechanically detached from the coverslips. Then, 100 µL of each well content was removed and serial dilutions were performed in NaCl 0.9% (w/v) and 20 µL of each dilution was plated in a CBA plate and incubated for 48 h at 37 °C and 10% CO_2_. The experiments were repeated at least three independent times.

### Biofilms assessed by LIVE/DEAD staining combined with confocal laser scanning microscopy

In order to evaluate the structure of the biofilm after the effect of *T. capitata* EOs and compounds, the biofilm was analyzed by confocal laser scanning microscopy (CLSM), as previously described^49^. Briefly, the biofilm of 4 *Gardnerella* species was performed in 24-well plates with coverslips, as described above and the conditions EO Car. at 0.16 µL/mL, and the combinations of 0.08 µL/mL carvacrol + 0.32 µL/mL linalool and 0.04 µL/mL carvacrol + 2.5 µL/mL ρ-cymene + 1.25 µL/mL linalool were tested. After 72 h, the biofilm was washed with NaCl 0.9% (w/v) and then stained using a LIVE/DEAD™ BacLight™ Bacterial Viability Kit (Invitrogen, Thermo Fisher Scientific), consisting of SYTO 9 and propidium iodide (PI). After incubation, the biofilms coating the coverslips were gently washed with 1 × PBS and then, the coverslips were removed from the wells and placed on microscope glass slides (VWR, Lisbon, Portugal). Two types of controls represented by untreated and dead biofilm cells were considered for this experiment and were used to define the CLSM laser intensity threshold. The dead control was obtained by covering the coverslips with the biofilms with 200 µL of 100% (v/v) methanol (Thermo Fisher Scientific) for 30 min. Then, all the coverslips with the live, dead, and treated biofilms were covered with 100 µL of the LIVE/ DEAD staining mix, with SYTO 9 and PI used each in a concentration of 3 µL/mL. Subsequently, the coverslips were incubated for 15 min. in the dark at room temperature. Biofilm image stacks were acquired with an Olympus™ FluoView FV1000 (Olympus, Lisbon, Portugal) confocal laser scanning microscope, using a 10 × objective. SYTO9 was detected using a filter with an excitation wavelength of 485 nm and an emission filter of 498 nm. PI was detected using a filter with an excitation wavelength of 536 nm and an emission filter of 617 nm. The CLSM images were analyzed using the FV10-ASW 4.0 Viewer Software (Olympus). This experiment was performed twice with two technical duplicates.

### LIVE/DEAD quantification of biofilm cells by fluorometry

To analyze the biofilm cells viability after the action of the compounds and EO, a quantitative LIVE/DEAD analysis was performed of the 4 *Gardnerella* species biofilm challenged with EO Car. at 0.16 µL/mL, and the combinations of 0.08 µL/mL carvacrol + 0.32 µL/mL linalool and 0.04 µL/mL carvacrol + 2.5 µL/mL ρ-cymene + 1.25 µL/mL linalool. After 72 h of biofilm formation as described above, the medium was removed, and the biofilm was washed with NaCl 0.9% (w/v) and then detached from the 24 well-plate using NaCl 0.85% (w/v) and centrifuged for 5 min at maximum velocity. Subsequently, the concentration of biofilm suspension was adjusted to 10^7^ CFU/mL and the LIVE/DEAD staining was added to biofilms as described in the CLSM section. Afterwards, the absorbance was measured using a multi-label microplate reader (Cytation 3, Bio Tek, Maine, USA) using the wavelengths of 485/530 nm and 485/630 nm (excitation/emission wavelength). Of note that before these experiments, a calibration curve was performed for each *Gardnerella* spp. according to the manufacturer’s instructions in each independent assay. In brief, from a fresh bacterial suspension collected from a CBA plate, suspensions with live and dead cells were prepared, as well as artificial dilutions of live/dead cells (33% live + 67% dead cells; 50% live + 50% dead cells; 67% live + 33% dead cells). A linear equation was obtained from the relation between the percentage of live/dead bacteria from the suspension and the green/red fluorescence ratio. Finally, for each biofilm assay, the obtained LIVE/DEAD ratios were then corrected according to the equation obtained from the calibration of the respective experiment. The percentage of reduction in cell integrity was obtained by comparison to the live control. Three independent assays were performed with two technical replicates.

### Biofilm cells recovery after removing the effect of EO and compounds from *T. capitata*

In an attempt to verify if biofilm cells can recover culturability after the activity of EOs and compounds, an experiment was performed as described for quantification of biofilm cells viability, in 24-well plates, without coverslips. After 48 h of biofilm formation, the medium was removed, and 1 mL of compounds was added. After 24 h of action of compounds and EOs on the biofilm, the medium from the biofilm was removed and the biofilm was washed with 1 mL of NaCl 0.9% (w/v). Then, 1 mL of fresh sBHI or sBHI supplemented with 0.25% (w/v) of maltose (Fisher Bioreagents, Fair Lawn, New Jersey, USA) was added to each well, and the plate was incubated for more 24 h at 37 °C and 10% CO_2_. After this time, the medium was removed, biofilm was washed again with NaCl 0.9% (w/v) and mechanically detached from the plate in 300 µL of sBHI. Serial dilutions in NaCl 0.9% (w/v) were performed and 20 µL of each dilution were plated in a CBA and incubated for 48 h at 37 °C and 10% CO_2_. The experiment was performed four independent times.

### Colonization of human vaginal epithelial tissue and activity of EO and compounds from *T. capitata*

To mimic the vaginal epithelial environment, a Reconstructed Human Vaginal Epithelium (HVE-SkinEthic®, Episkin, France) was used. A 24 h inoculum of *G. vaginalis* UM137 was prepared in sBHI medium. After 24 h the inoculum was centrifuged for 20 min, 3134 g, and the pellet was resuspended in a medium simulating genital tract secretions (mGTS)^100^. After that, the OD was adjusted to a concentration of 10^7^ CFU/mL and 1 mL was dispensed on the tissues for colonization of the vaginal epithelium. The tissues were then incubated at 37˚C and 10% CO_2_, for 9 h. Then, mGTS with either EO Car. at a concentration of 0.16 μL/mL or a combination of 0.08 μL/mL carvacrol + 0.32 μL/mL linalool was added to each tissue, after removal of the spent media, and incubated for a further 14 h. For microscopic analysis, the tissues were placed in 4% paraformaldehyde (Thermo Fisher Scientific) and then embedded in paraffin (Leica TP1020, Leica Biosystems, Nussloch, Germany). Paraffin tissue blocks were prepared (Leica EG 1140 H, Leica Biosystems, Nussloch, Germany) and 3-μm-thick sections were obtained using a microtome (Microm HM 325, Thermo Fisher Scientific, Walldorf, Germany). For the deparaffinization step, sections were placed in xylene (Thermo Fisher Scientific) twice for 5 min, followed by a hydration step with 100% and 50% of ethanol (Thermo Fisher Scientific) for 5 min each and a final step in distilled water for 5 min. The samples were allowed to air-dry and the PNA-FISH procedure was performed using a previously developed PNA probe for *Gardnerella*^101^, with a hybridization step at 60 °C for 90 min. The samples were analyzed using an Olympus BX51 epifluorescence microscope (Olympus, Lisbon, Portugal) equipped with a TRITC filter (BP 530–550, FT 570, LP 591 sensitive to the Alexa Fluor 594 molecule). To perform the culturability assays the tissues were washed once with NaCl 0.9% (w/v) and 500 µL of mGTS was added. A cycle of sonication was performed to displace the cells from the tissues, for 10 s with an amplitude of 33%. Serial dilutions in NaCl 0.9% (w/v) were then performed in duplicates from the content of each well, and each dilution was plated on CBA plates and incubated at 37ºC and 10% CO_2_, for 72 h. The experiment was performed twice, with technical duplicates.

### Vaginal irritation test

A toxicity study was performed using the Reconstructed Human Vaginal Epithelium (HVE-SkinEthic®, Episkin, France) model. This reconstructed epithelium reveals a strong histologic resemblance with human vaginal tissue. Upon arrival, the tissues were incubated overnight, at 37 °C, ≥ 90% humidity, 5% CO_2_ (Binder APT.lineTM C150E2 Incubator, Binder, NY, USA), in 6-well plates (VWR) using 1 mL of Maintenance Medium (provided by Episkin together with the tissues). Tissues were then transferred to new 24-well plates (one plate per condition) containing 300 μL of Maintenance Medium, and then 30 μL of the test substances or controls were gently dispersed over the entire tissue surface. EO Car. at 0.32 μL/mL and 0.08 μL/mL carvacrol + 0.32 μL/mL linalool tested concentrations were prepared in sesame oil (Ph Eur. Grade, Sigma), since this is the oily vehicle recommended for skin irritation testing according to ISO 10993-23:2021. Phosphate Buffer Saline without Ca^2+^ and Mg^2+^ (DPBS, VWR) and Sodium Lauryl Sulfate 1% w/v, were used as negative and positive controls, respectively. Solvent control (sesame oil) was also included. A different plate was used for each study substance (n = 3 tissues per substance). After 24 h of incubation at 37 °C, ≥ 90% humidity, 5% CO_2_, tissue integrity was assessed by the MTT assay, as previously described^102^. Briefly, the tissues were washed with PBS and gently dried. Then, the tissues were transferred to a 24-well plate containing 300 µL per well of a 0.5 mg/mL MTT (Alfa Aeser) solution (in PBS, VWR) and incubated for 3 h, at 37 °C, ≥ 90% humidity, 5% CO_2_, protected from light. After this period, the tissues were transferred to a single 24-well plate containing 750 µL of isopropyl alcohol and more 750 µL were further added at the top of each tissue to allow for the extraction of formazan for ≥ 2 h, in a sealed plastic bag, under agitation in a plate stirrer. Absorbance was then measured at 570 nm without a reference filter, according to the tissues provider instructions (Promega GloMax® Explorer System, USA). The background was deducted from all measured absorbance using isopropyl alcohol in free wells of the same reading plate. The absorbance of the negative control was considered 100% viability reference for products toxicity calculation.

### Statistical analysis

Data were analyzed with a one-way analysis of variance (ANOVA) and t-test with an alpha level of 0.05, using GraphPad Prism version 8 (GraphPad Software, California, USA). Results are presented as the mean + standard deviation (s.d.) or mean + standard error of the mean (s.e.m.). Statistical differences were considered significant when p-values were less than 0.05.

## Supplementary Information

Supplementary Figure 1.

## Data Availability

All data generated or analyzed during this study are included in this published article (and its Supplementary Information files).

## References

[1] Peebles K, Velloza J, Balkus JE, McClelland RS, Barnabas RV (2019). High global burden and costs of bacterial vaginosis: A systematic review and meta-analysis. Sex. Transm. Dis..

[2] Sobel JD (2000). Bacterial vaginosis. Annu. Rev. Med..

[3] Livengood CH (2009). Bacterial vaginosis: An overview for 2009. Rev. Obstet. Gynecol..

[4] Svare JA, Schmidt H, Hansen BB, Lose G (2006). Bacterial vaginosis in a cohort of Danish pregnant women: Prevalence and relationship with preterm delivery, low birthweight and perinatal infections. BJOG An Int. J. Obstet. Gynaecol..

[5] Isik G, Demirezen Ş, Dönmez H, Beksaç M (2016). Bacterial vaginosis in association with spontaneous abortion and recurrent pregnancy losses. J. Cytol..

[6] Ness RB (2005). A cluster analysis of bacterial vaginosis-associated microflora and pelvic inflammatory disease. Am. J. Epidemiol..

[7] Atashili J, Poole C, Ndumbe PM, Adimora AA, Smith JS (2008). Bacterial vaginosis and HIV acquisition: A meta-analysis of published studies. AIDS.

[8] Nouioui I (2018). Genome-based taxonomic classification of the phylum *actinobacteria*. Front. Microbiol..

[9] Ceccarani C (2019). Diversity of vaginal microbiome and metabolome during genital infections. Sci. Rep..

[10] Onderdonk AB, Delaney ML, Fichorova RN (2016). The human microbiome during bacterial vaginosis. Clin. Microbiol. Rev..

[11] Swidsinski A (2005). Adherent biofilms in bacterial vaginosis. Obstet. Gynecol..

[12] Muzny CA (2019). An updated conceptual model on the pathogenesis of bacterial vaginosis. J. Infect. Dis..

[13] Rosca AS, Castro J, Sousa LGV, Cerca N (2020). *Gardnerella* and vaginal health: the truth is out there. FEMS Microbiol. Rev..

[14] Hickey RJ, Forney LJ (2014). *Gardnerella vaginalis* does not always cause bacterial vaginosis. J. Infect. Dis..

[15] Vaneechoutte M (2019). Emended description of *Gardnerella vaginalis* and description of *Gardnerella leopoldii* sp. nov., *Gardnerella piotii* sp. nov. and *Gardnerella swidsinskii* sp. nov., with delineation of 13 genomic species within the genus *Gardnerella*. Int. J. Syst. Evol. Microbiol..

[16] Castro J (2013). Reciprocal interference between *Lactobacillus* spp. and *Gardnerella vaginalis* on initial adherence to epithelial cells. Int. J. Med. Sci..

[17] Schwebke JR, Muzny CA, Josey WE (2014). Role of *Gardnerella vaginalis* in the pathogenesis of bacterial vaginosis: A conceptual model. J. Infect. Dis..

[18] Workowski KA (2021). Sexually transmitted infections treatment guidelines, 2021. MMWR Recomm. Rep..

[19] Vodstrcil LA, Muzny CA, Plummer EL, Sobel JD, Bradshaw CS (2021). Bacterial vaginosis: Drivers of recurrence and challenges and opportunities in partner treatment. BMC Med..

[20] Ferris MJ (2004). Association of *Atopobium vaginae*, a recently described metronidazole resistant anaerobe, with bacterial vaginosis. BMC Infect. Dis..

[21] Beigi RH, Austin MN, Meyn LA, Krohn MA, Hillier SL (2004). Antimicrobial resistance associated with the treatment of bacterial vaginosis. Am. J. Obstet. Gynecol..

[22] Swidsinski A (2008). An adherent *Gardnerella vaginalis* biofilm persists on the vaginal epithelium after standard therapy with oral metronidazole. Am. J. Obstet. Gynecol..

[23] Muzny CA, Kardas P (2020). A narrative review of current challenges in the diagnosis and management of bacterial vaginosis. Sex. Transm. Dis..

[24] Machado D, Castro J, Palmeira-de-Oliveira A, Martinez-de-Oliveira J, Cerca N (2016). Bacterial vaginosis biofilms: Challenges to current therapies and emerging solutions. Front. Microbiol..

[25] Sobel JD, Sobel R (2021). Current and emerging pharmacotherapy for recurrent bacterial vaginosis. Expert Opin. Pharmacother..

[26] Falconi-McCahill A (2019). Bacterial vaginosis: A clinical update with a focus on complementary and alternative therapies. J. Midwifery Womens. Health.

[27] Burt S (2004). Essential oils: Their antibacterial properties and potential applications in foods: A review. Int. J. Food Microbiol..

[28] Hammer KA, Carson CF, Riley TV (1999). Antimicrobial activity of essential oils and other plant extracts. J. Appl. Microbiol..

[29] Khan MSA, Malik A, Ahmad I (2012). Anti-candidal activity of essential oils alone and in combination with amphotericin B or fluconazole against multi-drug resistant isolates of *Candida albicans*. Med. Mycol..

[30] Bozin B, Mimica-Dukic N, Samojlik I, Jovin E (2007). Antimicrobial and Antioxidant Properties of Rosemary and Sage (*Rosmarinus officinalis* L. and *Salvia officinalis* L., Lamiaceae) Essential Oils. J. Agric. Food Chem..

[31] Garozzo A (2009). In vitro antiviral activity of *Melaleuca alternifolia* essential oil. Lett. Appl. Microbiol..

[32] Hammer KA, Carson CF, Riley TV (1999). In vitro susceptibilities of lactobacilli and organisms associated with bacterial vaginosis to *Melaleuca alternifolia* (tea tree) oil. Antimicrob. Agents Chemother..

[33] Pina-Vaz C (2004). Antifungal activity of *Thymus* oils and their major compounds. J. Eur. Acad. Dermatol. Venereol..

[34] Karaman M (2017). *Origanum vulgare* essential oil affects pathogens causing vaginal infections. J. Appl. Microbiol..

[35] Hyldgaard M, Mygind T, Meyer RL (2012). Essential oils in food preservation: Mode of action, synergies, and interactions with food matrix components. Front. Microbiol..

[36] Barata AM, Rocha F, Lopes V, Bettencourt E, Figueiredo AC, Ozturk M, Ameenah G-FB (2011). Medicinal and aromatic plants: Portugal. Medicinal and Aromatic Plants of the World.

[37] da Cunha AP, Nogueira MT, Roque OR (2012). Plantas Aromáticas e Óleos Essenciais Composição e Aplicações.

[38] Raja RR (2012). medicinally potential plants of labiatae (Lamiaceae) family: An overview. Res. J. Med. Plant.

[39] Carapeto, A. *et al.* Lamiaceae: Espécies. *Flora-On: Flora de Portugal Interactiva, Sociedade Portuguesa de Botânica*. http://www.flora-on.pt/ (2022).

[40] Figueiredo AC (2008). Portuguese *Thymbra* and *Thymus* species volatiles: Chemical composition and biological activities. Curr. Pharm. Des..

[41] Palmeira-de-Oliveira A (2012). The anti-*Candida* activity of *Thymbra capitata* essential oil: Effect upon pre-formed biofilm. J. Ethnopharmacol..

[42] Neves A (2017). Characterization of Portuguese *Thymbra capitata*, *Thymus caespititius* and *Myrtus communis* essential oils in topical formulations. Flavour Fragr. J..

[43] Benbelaïd F (2014). Antimicrobial activity of some essential oils against oral multidrug-resistant *Enterococcus faecalis* in both planktonic and biofilm state. Asian Pac. J. Trop. Biomed..

[44] Miguel MG (2015). Antioxidant and antiproliferative activities of the essential oils from *Thymbra capitata* and *Thymus* Species grown in Portugal. Evid. Based. Complement. Alternat. Med..

[45] Machado D (2017). *Thymbra capitata* essential oil as potential therapeutic agent against *Gardnerella vaginalis* biofilm-related infections. Future Microbiol..

[46] Aljaafari MN (2021). An overview of the potential therapeutic applications of essential oils. Molecules.

[47] Basavegowda N, Baek K-H (2021). Synergistic antioxidant and antibacterial advantages of essential oils for food packaging applications. Biomolecules.

[48] Chouhan S, Sharma K, Guleria S (2017). Antimicrobial activity of some essential oils-present status and future perspectives. Medicines.

[49] Carvalhais V (2018). Tetracycline and rifampicin induced a viable but nonculturable state in *Staphylococcus epidermidis* biofilms. Future Microbiol..

[50] Giurazza R (2021). Emerging treatment options for multi-drug-resistant bacterial infections. Life.

[51] De Backer E (2006). Antibiotic susceptibility of *Atopobium vaginae*. BMC Infect. Dis..

[52] Austin MN, Beigi RH, Meyn LA, Hillier SL (2005). Microbiologic response to treatment of bacterial vaginosis with topical clindamycin or metronidazole. J. Clin. Microbiol..

[53] Li T (2020). Antimicrobial susceptibility testing of metronidazole and clindamycin against *Gardnerella vaginalis* in planktonic and biofilm formation. Can. J. Infect. Dis. Med. Microbiol..

[54] Tomás M, Palmeira-de-Oliveira A, Simões S, Martinez-de-Oliveira J, Palmeira-de-Oliveira R (2020). Bacterial vaginosis: Standard treatments and alternative strategies. Int. J. Pharm..

[55] Pietrella D (2011). Beneficial effect of *Mentha suaveolens* essential oil in the treatment of vaginal candidiasis assessed by real-time monitoring of infection. BMC Complement. Altern. Med..

[56] Mondello F, De Bernardis F, Girolamo A, Cassone A, Salvatore G (2006). *In vivo* activity of terpinen-4-ol, the main bioactive component of *Melaleuca alternifolia* Cheel (tea tree) oil against azole-susceptible and -resistant human pathogenic *Candida* species. BMC Infect. Dis..

[57] Trinh H-T, Lee I-A, Hyun Y-J, Kim D-H (2011). Artemisia princeps Pamp. essential oil and its constituents eucalyptol and α-terpineol ameliorate bacterial vaginosis and vulvovaginal candidiasis in mice by inhibiting bacterial growth and NF-κB activation. Planta Med..

[58] Masoudi M, Miraj S, Rafieian-Kopaei M (2016). Comparison of the effects of *Myrtus Communis L*, *Berberis Vulgaris* and metronidazole vaginal gel alone for the treatment of bacterial vaginosis. J. Clin. Diagn. Res..

[59] Abdali K (2015). Comparison of the effect of vaginal *Zataria multiflora* cream and oral metronidazole pill on results of treatments for vaginal infections including trichomoniasis and bacterial vaginosis in women of reproductive age. Biomed. Res. Int..

[60] Schwiertz A, Duttke C, Hild J, Müller HJ (2006). In vitro activity of essential oils on microorganisms isolated from vaginal infections. Int. J. Aromather..

[61] Demuner AJ (2011). Seasonal variation in the chemical composition and antimicrobial activity of volatile oils of three species of *Leptospermum* (Myrtaceae) grown in Brazil. Molecules.

[62] Castro J, Salgueiro L, Cerca N, Singh S (2021). Essential oils as potential antibiofilm agents: Insights into the key role of thymbra capitata to fight biofilm-associated infections. Volatile Oils: Production, Composition and Uses.

[63] Dorman HJ, Deans SG (2000). Antimicrobial agents from plants: Antibacterial activity of plant volatile oils. J. Appl. Microbiol..

[64] Guimarães AC (2019). Antibacterial activity of terpenes and terpenoids present in essential oils. Molecules.

[65] Casiglia S, Bruno M, Scandolera E, Senatore F, Senatore F (2019). Influence of harvesting time on composition of the essential oil of *Thymus capitatus* (L.) Hoffmanns. & Link. growing wild in northern Sicily and its activity on microorganisms affecting historical art crafts. Arab. J. Chem..

[66] Ultee A, Slump RA, Steging G, Smid EJ (2000). Antimicrobial activity of carvacrol toward *Bacillus cereus* on rice. J. Food Prot..

[67] Bagamboula C, Uyttendaele M, Debevere J (2004). Inhibitory effect of thyme and basil essential oils, carvacrol, thymol, estragol, linalool and p-cymene towards *Shigella sonnei* and *S. flexneri*. Food Microbiol..

[68] Merghni A (2018). Assessment of the antibiofilm and antiquorum sensing activities of *Eucalyptus globulus* essential oil and its main component 1,8-cineole against methicillin-resistant *Staphylococcus aureus* strains. Microb. Pathog..

[69] Herman A, Tambor K, Herman A (2016). Linalool affects the antimicrobial efficacy of essential oils. Curr. Microbiol..

[70] Bassolé IHN, Juliani HR (2012). Essential oils in combination and their antimicrobial properties. Molecules.

[71] Rattanachaikunsopon P, Phumkhachorn P (2010). Assessment of factors influencing antimicrobial activity of carvacrol and cymene against *Vibrio cholerae* in food. J. Biosci. Bioeng..

[72] Periago PM, Delgado B, Fernández PS, Palop A (2004). Use of carvacrol and cymene to control growth and viability of *Listeria monocytogenes* cells and predictions of survivors using frequency distribution functions. J. Food Prot..

[73] Kiskó G, Roller S (2005). Carvacrol and p-cymene inactivate *Escherichia coli* O157:H7 in apple juice. BMC Microbiol..

[74] Rafaque Z (2020). In-vitro investigation of antibiotics efficacy against uropathogenic *Escherichia coli* biofilms and antibiotic induced biofilm formation at sub-minimum inhibitory concentration of ciprofloxacin. Infect. Drug Resist..

[75] Lagha R, Abdallah FB, Al-Sarhan BO, Al-Sodany Y (2019). Antibacterial and biofilm inhibitory activity of medicinal plant essential oils against *Escherichia coli* isolated from UTI patients. Molecules.

[76] Borges A, Lopez-Romero JC, Oliveira D, Giaouris E, Simões M (2017). Prevention, removal and inactivation of *Escherichia coli* and *Staphylococcus aureus* biofilms using selected monoterpenes of essential oils. J. Appl. Microbiol..

[77] Pasquaroli S (2013). Antibiotic pressure can induce the viable but non-culturable state in *Staphylococcus aureus* growing in biofilms. J. Antimicrob. Chemother..

[78] Bradshaw CS (2006). High recurrence rates of bacterial vaginosis over the course of 12 months after oral metronidazole therapy and factors associated with recurrence. J. Infect. Dis..

[79] Bradshaw CS (2006). The association of *Atopobium vaginae* and *Gardnerella vaginalis* with bacterial vaginosis and recurrence after oral metronidazole therapy. J. Infect. Dis..

[80] Alves CT (2014). *Candida albicans* promotes invasion and colonisation of *Candida glabrata* in a reconstituted human vaginal epithelium. J. Infect..

[81] Stabile G, Ricci G, Scalia MS, Seta FD (2021). Induced dryness stress on human vaginal epithelium: The efficacy of a new vaginal gel. Gels.

[82] Verstraelen H, Swidsinski A (2013). The biofilm in bacterial vaginosis. Curr. Opin. Infect. Dis..

[83] Nafis A (2021). New insight into the chemical composition, antimicrobial and synergistic effects of the moroccan endemic *Thymus atlanticus* (Ball) roussine essential oil in combination with conventional antibiotics. Molecules.

[84] Azeredo J (2017). Critical review on biofilm methods. Crit. Rev. Microbiol..

[85] Liu X (2020). Antibacterial activity and mechanism of linalool against *Pseudomonas aeruginosa*. Microb. Pathog..

[86] Guo F (2021). Antimicrobial activity and proposed action mechanism of linalool against *Pseudomonas fluorescens*. Front. Microbiol..

[87] Yang S-K (2021). Combinatorial antimicrobial efficacy and mechanism of linalool against clinically relevant *Klebsiella pneumoniae*. Front. Microbiol..

[88] Council of Europe. *European Pharmacopoeia*. (European Directorate for the Quality of Medicines and Healthcare of the Council of Europe, 2010).

[89] Cavaleiro C, Salgueiro LR, Miguel MG, Da Cunha AP (2004). Analysis by gas chromatography-mass spectrometry of the volatile components of *Teucrium lusitanicum* and *Teucrium algarbiensis*. J. Chromatogr. A.

[90] Wallace, W. E. Retention Indices. in *NIST Chemistry WebBook, NIST Standard Reference Database Number 69* (eds. Linstrom, P. J. & Mallard, W. G.) (National Institute of Standards and Technology, 2022).

[91] McLafferty FW (2010). Wiley Registry of Mass Spectral Data.

[92] Adams RP (2007). Identification of Essential Oil Components by Gas Chromatography/Mass Spectrometry.

[93] Castro J (2015). Using an *in-vitro* biofilm model to assess the virulence potential of bacterial vaginosis or non-bacterial vaginosis *Gardnerella vaginalis* isolates. Sci. Rep..

[94] Castro J, Rosca AS, Cools P, Vaneechoutte M, Cerca N (2020). *Gardnerella vaginalis* enhances *Atopobium vaginae* viability in an in vitro model. Front. Cell. Infect. Microbiol..

[95] Climo MW, Patron RL, Archer GL (1999). Combinations of vancomycin and beta-lactams are synergistic against staphylococci with reduced susceptibilities to vancomycin. Antimicrob. Agents Chemother..

[96] Bonapace CR, Bosso JA, Friedrich LV, White RL (2002). Comparison of methods of interpretation of checkerboard synergy testing. Diagn. Microbiol. Infect. Dis..

[97] Marques MB, Brookings ES, Moser SA, Sonke PB, Waites KB (1997). Comparative in vitro antimicrobial susceptibilities of nosocomial isolates of *Acinetobacter baumannii* and synergistic activities of nine antimicrobial combinations. Antimicrob. Agents Chemother..

[98] Machado D, Palmeira-de-Oliveira A, Cerca N (2015). Optimization of culture conditions for *Gardnerella vaginalis* biofilm formation. J. Microbiol. Methods.

[99] Peeters E, Nelis HJ, Coenye T (2008). Comparison of multiple methods for quantification of microbial biofilms grown in microtiter plates. J. Microbiol. Methods.

[100] Stingley RL, Liu H, Mullis LB, Elkins CA, Hart ME (2014). *Staphylococcus aureus* toxic shock syndrome toxin-1 (TSST-1) production and *Lactobacillus species* growth in a defined medium simulating vaginal secretions. J. Microbiol. Methods.

[101] Machado A (2013). Fluorescence in situ Hybridization method using Peptide Nucleic Acid probes for rapid detection of *Lactobacillus* and *Gardnerella* spp. BMC Microbiol..

[102] Ayehunie S, Wang YY, Landry T, Bogojevic S, Cone RA (2018). Hyperosmolal vaginal lubricants markedly reduce epithelial barrier properties in a three-dimensional vaginal epithelium model. Toxicol. Rep..

