# Supplementary material for: Synergistic effects of carvacrol, α-terpinene, γ-terpinene, ρ-cymene and linalool against Gardnerella species
Source: Sci Rep. 2022 Mar 15;12:4417. doi: 10.1038/s41598-022-08217-w (PMC8924259; doi:10.1038/s41598-022-08217-w)
Supplement: Supplementary file 1 — Supplementary Figure 1. [file 41598_2022_8217_MOESM1_ESM.docx]

**Supplementary Information**

**Synergistic effects of Carvacrol, α-Terpinene, γ-Terpinene, ρ-Cymene and Linalool against *Gardnerella* species**

Lúcia G. V. Sousa, Joana Castro, Carlos Cavaleiro, Lígia Salgueiro, Mariana Tomás, Rita Palmeira-Oliveira, José Martinez-Oliveira, Nuno Cerca


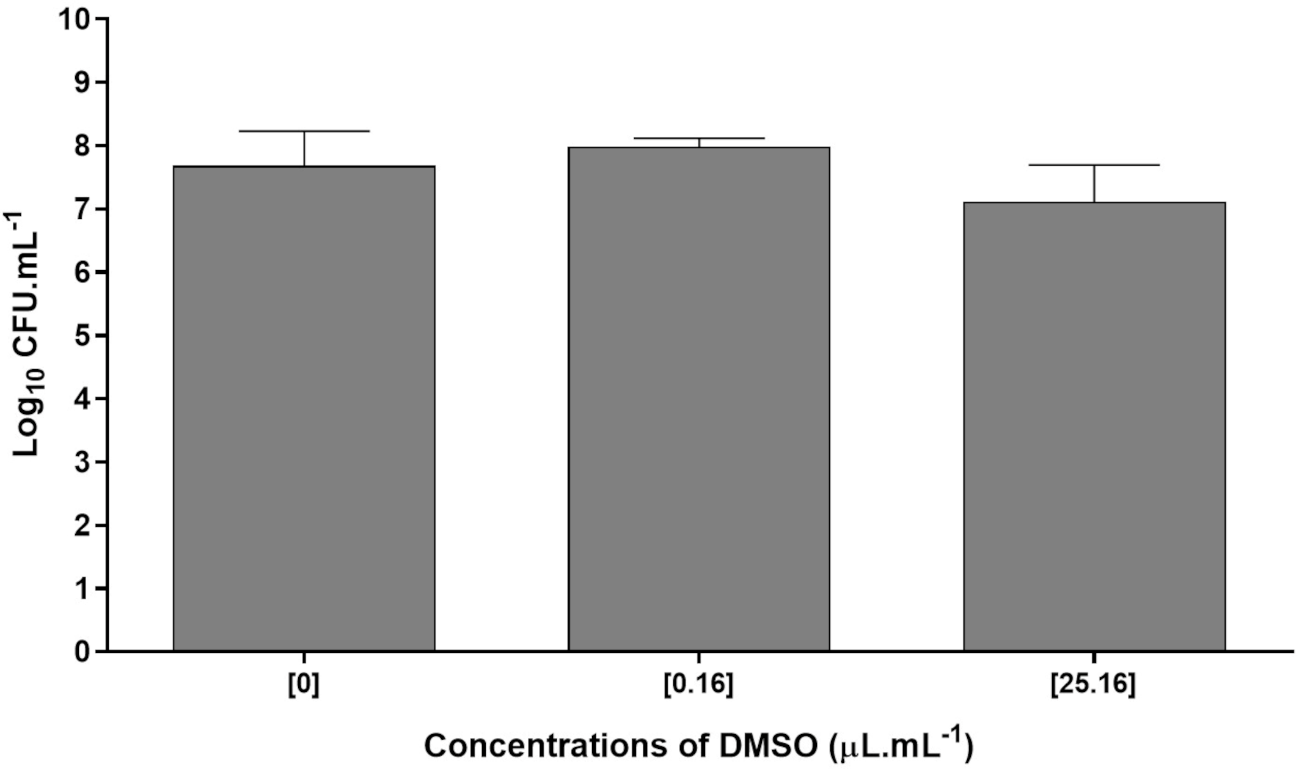


**Supplementary Figure S1:** Results of *Gardnerella* sp. UM241 biofilm cells culturability, after the application of DMSO at the lowest (0.16 µL/mL) and highest (25.16 µL/mL) concentrations used to dissolve the compounds. The results represent mean of Log CFU.mL^-1^ and error bars represent s.d. No statistical differences with the control were found when analyzing data using a t-test (n≥4).
